# FAK deletion accelerates liver regeneration after two-thirds partial hepatectomy

**DOI:** 10.1038/srep34316

**Published:** 2016-09-28

**Authors:** Na Shang, Maribel Arteaga, Lennox Chitsike, Fang Wang, Navin Viswakarma, Peter Breslin, Wei Qiu

**Affiliations:** 1 Departments of Surgery and Oncology Institute and Stritch School of Medicine, 2160 South 1^st^ Avenue, Maywood, IL 60153, USA; 2 Departments of Molecular/Cellular Physiology, Stritch School of Medicine, 2160 South 1^st^ Avenue, Maywood, IL 60153, USA; 3 Department of Biology, Loyola University Chicago, 1032 W. Sheridan Rd., Chicago, IL 60660, USA; 4 Department of Surgery, University of Illinois at Chicago 840 South Wood Street Chicago, IL 60612, USA

## Abstract

Understanding the molecular mechanisms of liver regeneration is essential to improve the survival rate of patients after surgical resection of large amounts of liver tissue. Focal adhesion kinase (FAK) regulates different cellular functions, including cell survival, proliferation and cell migration. The role of FAK in liver regeneration remains unknown. In this study, we found that Fak is activated and induced during liver regeneration after two-thirds partial hepatectomy (PHx). We used mice with liver-specific deletion of *Fak* and investigated the role of Fak in liver regeneration in 2/3 PHx model (removal of 2/3 of the liver). We found that specific deletion of *Fak* accelerates liver regeneration. *Fak* deletion enhances hepatocyte proliferation prior to day 3 post-PHx but attenuates hepatocyte proliferation 3 days after PHx. Moreover, we demonstrated that the deletion of *Fak* in liver transiently increases EGFR activation by regulating the TNFα/HB-EGF axis during liver regeneration. Furthermore, we found more apoptosis in *Fak*-deficient mouse livers compared to *WT* mouse livers after PHx. Conclusion: Our data suggest that *Fak* is involved in the process of liver regeneration, and inhibition of FAK may be a promising strategy to accelerate liver regeneration in recipients after liver transplantation.

Liver regeneration is a well-orchestrated and tightly regulated biological response to hepatocellular injury or loss involving a complex network of inflammatory, proliferative, and metabolic signals^1^. Liver regeneration is essential to improve the survival rate of patients after surgical resection of large amounts of liver tissue. In addition, the improvement of the liver regenerative process would reduce the amount of liver tissue required for liver transplantation. This will reduce the risk for the donor and would also enhance the growth of the transplant within the recipient.

Two-thirds partial hepatectomy (PHx) in rodents has become a useful paradigm for studying liver regeneration^2^. With this model, molecular mechanisms of liver regeneration have been emerging. Hepatocyte growth factor (HGF), a major hepatocyte mitogen, is important for liver regeneration through its activation of c-MET^3^,^4^. Activation of epidermal growth factor receptor (EGFR) signaling is required for efficient liver regeneration. Mice lacking the EGFR in the liver after PHx showed reduced hepatocyte proliferation and delayed liver regeneration, resulting from a defective entry into the G1–S phase of the cell cycle^5^. Consistently, a number of kinases that are targets of HGF/c-MET and EGFR signaling, such as protein kinase B (PKB or AKT)^6^, extracellular receptor kinase (ERK)^7^ or signal transducer and activator of transcription 3 (Stat3)^8^, also play important roles in liver regeneration.

Integrin pathways have been shown to function in the liver regenerative process. Knockdown and knockout of *β1-integrin* in hepatocytes impairs liver regeneration through inhibition of EGFR and c-MET activation^9^, suggesting that integrin signaling is required for liver regeneration. However, deletion of integrin-linked kinase (ILK), an important downstream target of integrin, enhances liver regeneration and enlarges liver mass after PHx^10^. Focal adhesion kinase (FAK) is another important protein involved in the transmission of integrin signals^11^,^12^. Activation of FAK can target multiple downstream signaling pathways (*e.g.*, AKT, ERK and Ras-related C3 botulinum toxin substrate (Rac)), thereby regulating different cellular functions, including cell survival, proliferation and migration^13^. We have recently shown that FAK is required for c-MET/β-catenin-induced hepatocarcinogenesis by activation of AKT and ERK^14^. Since FAK is important in cell proliferation, it is reasonable to suspect that a role exists for FAK in liver regeneration. However, such a role has as yet to be determined.

In this study, we investigated the role of FAK in liver regeneration in 2/3 PHx model (resection of two-thirds of the mass of liver tissue). Interestingly, we found that specific deletion of *Fak* in mouse liver accelerates liver regeneration after 2/3 PHx. Consistently, *Fak* deletion enhances hepatocyte proliferation prior to day 3 after PHx but attenuates hepatocyte proliferation 3 days after PHx. Intriguingly, we found that the deletion of *Fak* in mouse liver significantly increases EGFR activation but decreases c-MET activation during liver regeneration. Furthermore, we found that *Fak* deficiency increases HB-EGF (a ligand of EGFR) during liver regeneration and a specific HB-EGF inhibitor abrogates accelerated liver regeneration and enhanced EGFR activation after PHx. Moreover, we discovered that *Fak* deficiency increases TNFα expression after PHx and a neutralizing TNFα antibody suppresses accelerated liver regeneration, enhanced HB-EGF expression and EGFR activation in *Fak*-deficient mice. In addition, more apoptosis was found in *Fak*-deficient mouse livers compared to *WT* mouse livers after PHx. In general, our data suggest that *Fak* deletion accelerates the liver regenerative process by regulating the TNFα/HB-EGF/EGFR axis. Inhibition of FAK may be a promising strategy to accelerate liver regeneration in recipients following liver transplantation.

## Results

### FAK is activated and induced during liver regeneration after 2/3 PHx

**Figure 1:**
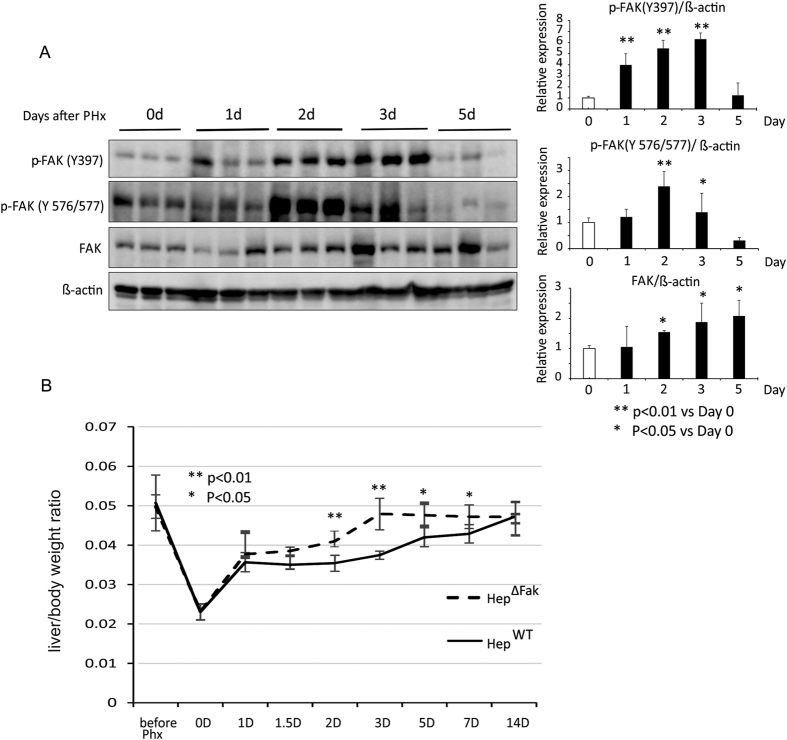
Deletion of *Fak* accelerates liver regeneration after PHx. (**A**) Left, expression of Fak protein, p-FAK (Y397), p-FAK (Y 576/577) and β-actin in whole livers of *WT* C57BL/6 mice 0, 1, 2, 3 and 5 days after PHx. Right, quantification of Western blotting by *Image J* software. **(B)** Liver weight/body weight ratio was analyzed in Alb-Cre (Hep^WT^) and Alb-Cre; Fak^flox/flox^ (Hep^∆Fak^) mice after PHx (5 mice per strain at each time point).

The kinase activity of FAK plays a critical role in its functions^15^,^16^. Phosphorylation of FAK on Tyr397 is required for its activation^17^,^18^. To study the role of FAK in liver regeneration, we first examined whether FAK is activated during liver regeneration after 2/3 PHx. We found that phosphorylation of FAK on Tyr397 was significantly increased in the livers of mice which underwent PHx 1 day post-surgery compared to the livers of control mice (Fig. 1A). The activation of FAK reached a peak on day 3 post-surgery and decreased to normal by day 5 (Fig. 1A). Interestingly, total FAK expression was also induced on days 2 and 3 after PHx and decreased to a normal level by day 5 (Fig. 1A). Our data indicate that FAK is induced and activated during liver regeneration.

### Deletion of *Fak* in mouse liver accelerates liver regeneration after 2/3 PHx

To study the role of FAK in liver regeneration, we performed 2/3 PHx on age- and gender-matched Alb-Cre (Hep^WT^) and Alb-Cre; Fak^flox/flox^ (Hep^∆Fak^) mice (Fig. 1B). Mice were sacrificed 1, 1.5, 2, 3, 5, 7 and 14 days after PHx and their livers were collected and analyzed. Intriguingly, we found that the liver regenerative process in Hep^∆Fak^ mice was significantly accelerated compared to Hep^WT^ mice (Fig. 1B). The relative liver weight versus body weight in Hep^∆Fak^ mice was increased by 20% compared to Hep^WT^ mice on post-surgical day 2 (Fig. 1B). Three days after PHx, the liver mass in Hep^∆Fak^ mice recovered to almost 100% while liver mass recovered to only 76% in Hep^WT^ mice (Fig. 1B). The liver mass in Hep^∆Fak^ mice did not continue to increase and remained at 100% 3 days after PHx, while the liver mass of Hep^WT^ mice continued to grow until reaching 100% 14 days after PHx. These data indicate that a deficiency of *Fak* in mouse liver accelerates liver regeneration after PHx.

### *Fak* deficiency in mouse liver accelerates hepatocyte proliferation during early liver regeneration after 2/3 PHx

**Figure 2:**
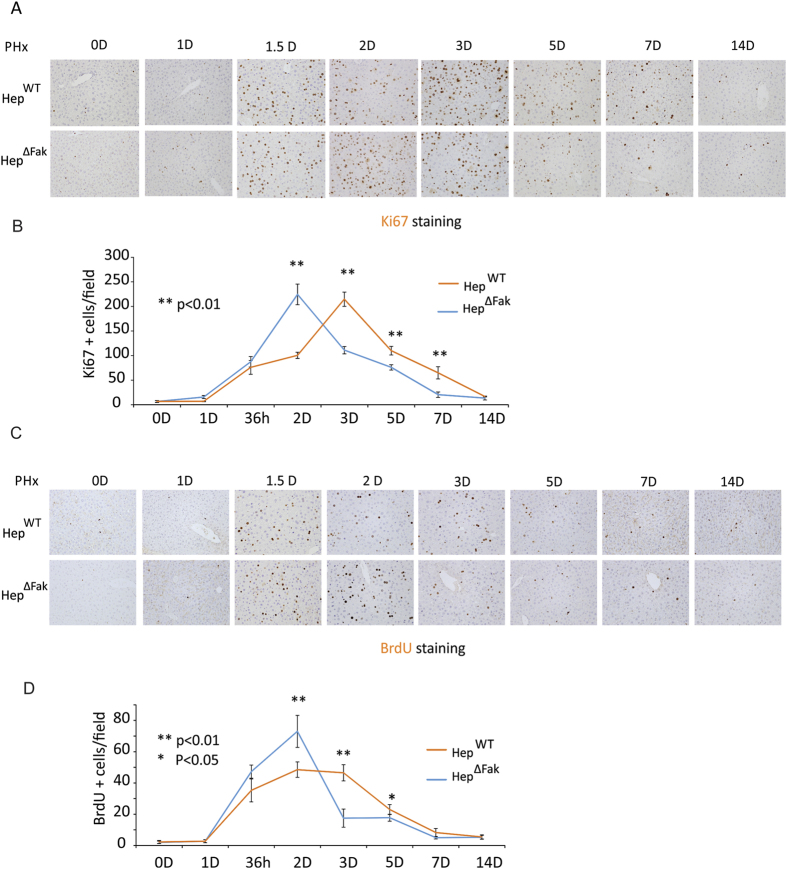
Deletion of *Fak* accelerates proliferation of hepatocytes after PHx. **(A)** Representative photomicrographs of immunohistochemistry for Ki67 in the livers of Hep^WT^ and Hep^∆Fak^ mice 0, 1, 1.5, 2, 3, 5, 7 and 14 days after PHx. **(B)** Quantification of Ki67 staining for **(A)** (n = 5). **(C)** Representative photomicrographs of immunohistochemistry for BrdU in the livers of Hep^WT^ and Hep^∆Fak^ mice 0, 1, 1.5, 2, 3, 5, 7 and 14 days after PHx. **(D)** Quantification of BrdU staining for **(C)** (n = 5).

Liver mass is replenished by the replication of hepatocytes^19^. Therefore, dramatically increased hepatocyte proliferation takes place during liver regeneration after PHx^2^. We therefore analyzed proliferation in the livers of Hep^WT^ and Hep^∆Fak^ mice using Ki67 and BrdU staining. The number of Ki67- and BrdU-positive cells was significantly increased in *Fak*-deficient livers compared to *WT* livers by day 2 after PHx (Fig. 2A–D). However, hepatocyte proliferation significantly decreased by day 3 in Hep^∆Fak^ mice while hepatocyte proliferation in Hep^WT^ mice reached a peak on day 3. Although hepatocyte proliferation in Hep^WT^ mice also declined after day 3, greater hepatocyte proliferation continued to be observed in Hep^WT^ livers compared to Hep^∆Fak^ livers even by day 7. These results demonstrate that *Fak* deficiency accelerates hepatocyte proliferation during liver regeneration.

### *Fak* deficiency in mouse liver accelerates liver regeneration by enhancing EGFR activation following 2/3 PHx

**Figure 3:**
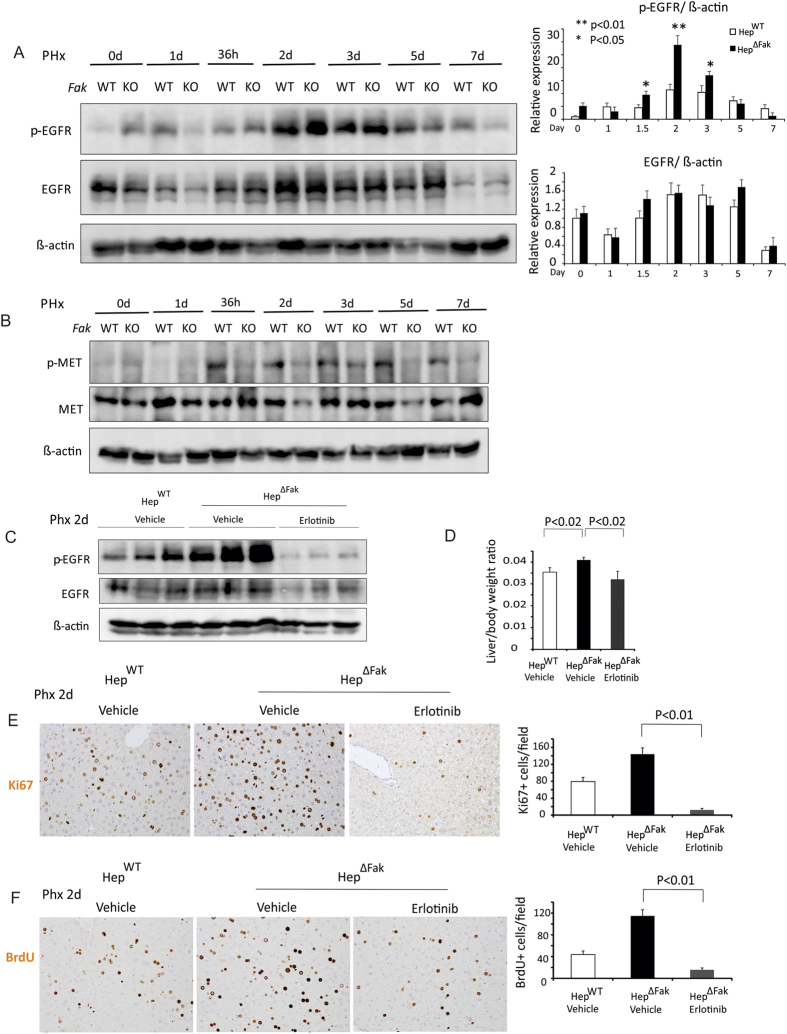
*Fak* deficiency accelerates proliferation of hepatocytes after PHx by enhancing activation of EGFR. **(A)** Left, expression of p-EGFR, EGFR and β-actin proteins in whole livers of Hep^WT^ and Hep^∆Fak^ mice (pooled samples from 3 mice) 0, 1, 1.5, 2, 3, 5 and 7 days after PHx. Right, quantification of western blotting by *Image J* software. **(B)** Expression of p-MET, MET and β-actin proteins in whole livers of Hep^WT^ and Hep^∆Fak^ mice (pooled samples from 3 mice) 0, 1, 1.5, 2, 3, 5 and 7 days after PHx. **(C)** Expression of p-EGFR, EGFR and β-actin proteins in whole livers of Hep^WT^ and Hep^∆Fak^ mice treated with either vehicle or 50 mg/kg erlotinib by oral gavage daily for 3 days beginning one day before PHx. **(D)** Liver weight/body weight ratios were analyzed in Hep^WT^ and Hep^∆Fak^ mice treated with either vehicle or 50 mg/kg erlotinib by oral gavage daily for 3 days beginning one day before PHx (n = 6). **(E)** Representative photomicrographs and quantification of immunohistochemistry for Ki67 in the livers of Hep^WT^ and Hep^∆Fak^ mice for **(D)** (n = 6). **(F)** Representative photomicrographs and quantification of immunohistochemistry for BrdU in the livers of Hep^WT^ and Hep^∆Fak^ mice for **(D)** (n = 6).

EGFR and HGF/c-MET signaling pathways play key roles in hepatocyte proliferation^3^,^4^,^5^. We therefore examined whether *Fak* deficiency in hepatocytes might affect EGFR and HGF/c-MET signaling. Intriguingly, we found that phosphorylation of EGFR, which leads to EGFR activation, is significantly enhanced in Hep^∆Fak^ mice compared to Hep^WT^ mice by 1.5–3 days after PHx (Fig. 3A). We did not find a significant difference in total EGFR expression between Hep^WT^ and Hep^∆Fak^ mice (Fig. 3A), suggesting that *Fak* deficiency enhances EGFR activation during liver regeneration. We also examined the effect of *Fak* deficiency on c-MET activation during liver regeneration. Interestingly, phosphorylation of c-MET (on Tyr 1234/1235) during liver regeneration, which causes activation of c-MET^20^, was significantly suppressed in *Fak*-deficient livers (Fig. 3B).

We have found that FAK mediates the activation of AKT and ERK induced by MET in HCC cells^14^. AKT and ERK could be also activated by EGFR activation^21^. Therefore, it would be interesting to see if activation of AKT or ERK is affected by *Fak* deficiency during liver regeneration. We examined phosphorylation of AKT and ERK in *WT* and *Fak*-KO mice after 2/3 PHX. We found that p-AKT and p-ERK were decreased in Hep^∆Fak^ mice compared to Hep^WT^ mice by 2–5 days after PHx (Fig. S1). These data suggest that *Fak* deficiency accelerates liver regeneration not by enhancement of AKT or ERK activation. STAT3, another downstream target of EGFR, has been reported to promote hepatocyte proliferation during liver regeneration^8^. We examined phosphorylation of STAT3 in WT and *Fak*-KO mice after 2/3 PHX. We found that phosphorylation of STAT3 was increased in Hep^∆Fak^ mice compared to Hep^WT^ mice by 1–2 days post-PHx. These data suggest that *Fak* deletion might accelerate liver regeneration by enhancing EGFR/STAT3 activation.

To further determine whether *Fak* deficiency accelerates liver regeneration by enhancing EGFR activation, we examined whether erlotinib, a specific EGFR inhibitor, attenuates liver regeneration in *Fak*-deficient mice. We treated *Fak*-deficient mice with 50 mg/kg erlotinib by oral gavage daily for 3 days starting one day prior to PHx. We found that erlotinib significantly attenuated EGFR activation (Fig. 3C), liver regeneration (Fig. 3D) and hepatocyte proliferation (Fig. 3E,F) in Hep^∆Fak^ mice. In general, these data indicate that *Fak* deficiency accelerates liver regeneration by enhancing EGFR activation.

### *Fak* deficiency in mouse liver enhances EGFR activation by increasing HB-EGF expression after 2/3 PHx

**Figure 4:**
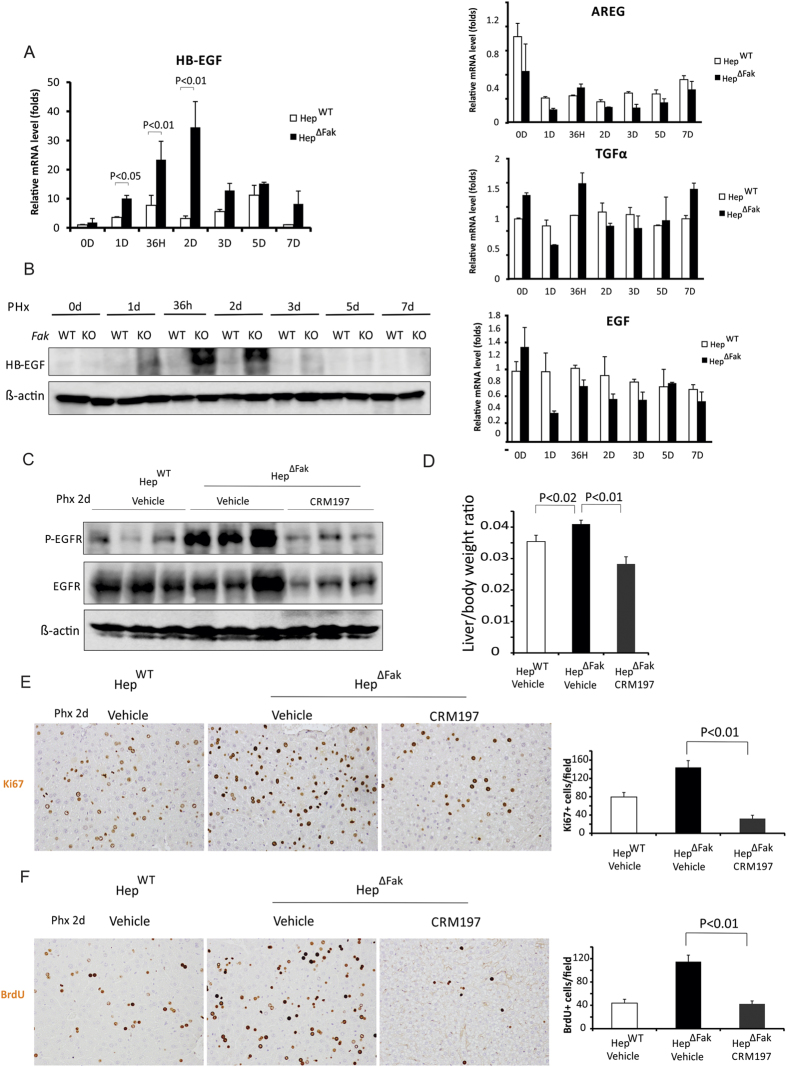
*Fak* deficiency increases EGFR activation and proliferation of hepatocytes after PHx by increasing expression of HB-EGF. **(A)** *HB-EGF*, *TGFα*, *EGF* and *AREG* mRNA expression levels in whole livers of Hep^WT^ and Hep^∆Fak^ mice 0, 1, 1.5, 2, 3, 5 and 7 days after PHx (n = 6). **(B)** HB-EGF protein expression levels in whole livers of Hep^WT^ and Hep^∆Fak^ mice (pooled samples from 3 mice) 0, 1, 1.5, 2, 3, 5 and 7 days after PHx (n = 6). **(C)** Expression of p-EGFR, EGFR and β-actin proteins in whole livers of Hep^WT^ and Hep^∆Fak^ mice treated with either vehicle or CRM197 by oral gavage daily for 3 days starting one day before PHx. **(D)** Liver weight/body weight ratios were analyzed in Hep^WT^ and Hep^∆Fak^ mice treated with either vehicle or CRM197 by oral gavage daily for 3 days starting one day before PHx (n = 6). (**E)** Representative photomicrographs and quantification of immunohistochemistry for Ki67 in the livers of Hep^WT^ and Hep^∆Fak^ mice (n = 6) for **(D)**. **(F)** Representative photomicrographs and quantification of immunohistochemistry for BrdU in the livers of Hep^WT^ and Hep^∆Fak^ mice (n = 6) for **(D)**.

Several EGFR ligands, including EGF, TGFα, heparin binding EGF (HB-EGF) and amphiregulin (ARG) have been shown to activate EGFR during liver regeneration^1^. We therefore examined whether *Fak* deficiency enhances those EGFR ligands during liver regeneration. Interestingly, we found that mRNA levels of HB-EGF, but not EGF, TGFα nor ARG, were rapidly enhanced by *Fak* deficiency in mouse livers after 2/3 PHx (Fig. 4A). We confirmed that protein levels of HB-EGF were also rapidly enhanced by *Fak* deficiency in mouse livers after 2/3 PHx (Fig. 4B). HB-EGF transgenic mice have enhanced hepatocyte proliferation during early liver regeneration while liver regeneration was delayed in HB-EGF-knockout mice after 2/3 PHx^22^. Therefore, *Fak* deficiency in hepatocytes may enhance EGFR activation through increasing HB-EGF expression after 2/3 PHx. To test this hypothesis, we treated *Fak*-deficient mice with CRM197, a specific inhibitor of HB-EGF^23^,^24^, daily for 3 days starting one day pre-PHx. We found that CRM197 significantly attenuated EGFR activation (Fig. 4C), liver regeneration (Fig. 4D) and hepatocyte proliferation (Fig. 4E,F) in Hep^∆Fak^ mice after PHx. These results suggest that *Fak* deficiency enhances EGFR activation and accelerates liver regeneration by increasing HB-EGF expression.

### *Fak* deficiency in mouse liver increases HB-EGF expression by enhancing tumor necrosis factor (TNFα) expression after 2/3 PHx

**Figure 5:**
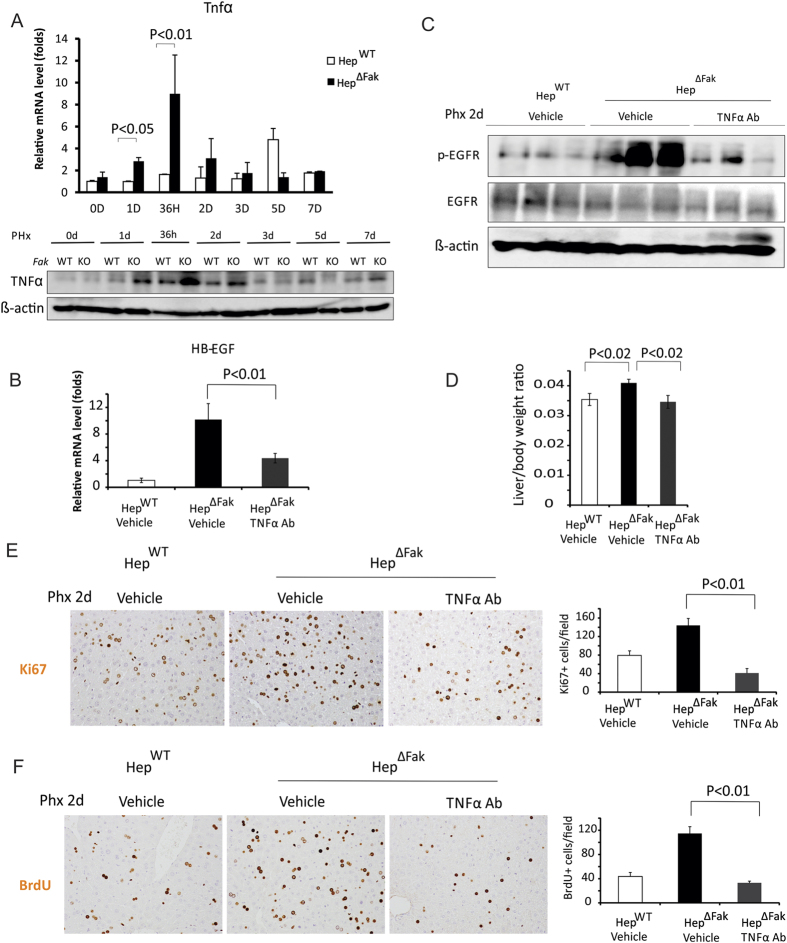
*Fak* deficiency increases HB-EGF and proliferation of hepatocytes after PHx by increasing the expression of TNFα. **(A)** TNFα mRNA (top) and protein (bottom) expression levels in the whole livers of Hep^WT^ and Hep^∆Fak^ mice 0, 1, 1.5, 2, 3, 5 and 7 days after PHx (n = 6). **(B)** HB-EGF mRNA expression level in whole livers of Hep^WT^ and Hep^∆Fak^ mice treated with either vehicle or a neutralized TNFα antibody by i.p. injection daily for 3 days starting one day prior to PHx. **(C)** expression of p-EGFR, EGFR and β-actin proteins in whole livers of Hep^WT^ and Hep^∆Fak^ mice treated with either vehicle or a neutralized TNFα antibody by oral gavage daily for 3 days starting one day before PHx. **(D)** Liver weight/body weight ratios were analyzed in the Hep^WT^ and Hep^∆Fak^ mice treated with either vehicle or a neutralized TNFα antibody by oral gavage daily for 3 days starting one day before PHx (n = 6). (**E)** Representative photomicrographs and quantification (n = 6) of immunohistochemistry for Ki67 in the livers of Hep^WT^ and Hep^∆Fak^ mice for **(D)**. **(E)** Representative photomicrographs and quantification of immunohistochemistry for BrdU in the livers of Hep^WT^ and Hep^∆Fak^ mice (n = 5) for **(D)**.

HB-EGF can be induced by TNFα in vascular endothelial cells^25^. Inhibition of TNFα signaling by TNFα-neutralizing antibodies or genetic deletion of *TNF receptor 1* reduced hepatocyte proliferation and liver regeneration^26^,^27^. We therefore hypothesized that *Fak* deficiency in mouse liver may increase TNFα, thereby enhancing HB-EGF expression. Indeed, we found that mRNA and protein level of TNFα were significantly enhanced by *Fak* deficiency in mouse livers during liver regeneration after 2/3 PHx (Fig. 5A,B). To examine whether increased TNFα in *Fak*-deficient livers enhances HB-EGF expression and liver regeneration, we treated *Fak*-deficient mice with a TNFα-neutralizing antibody daily for 2 days starting on one day prior to PHx. We found that the TNFα-neutralizing antibody significantly suppressed *HB-EGF* mRNA expression (Fig. 5B), EGFR activation (Fig. 5C), liver regeneration (Fig. 5D) and hepatocyte proliferation (Fig. 5E,F) in Hep^∆Fak^ mice. These results indicate that *Fak* deficiency enhances *HB-EGF* expression, EGFR activation and accelerates liver regeneration by increasing TNFα expression.

### *Fak* deficiency in mouse liver increases death of hepatocytes after 2/3 PHx

**Figure 6:**
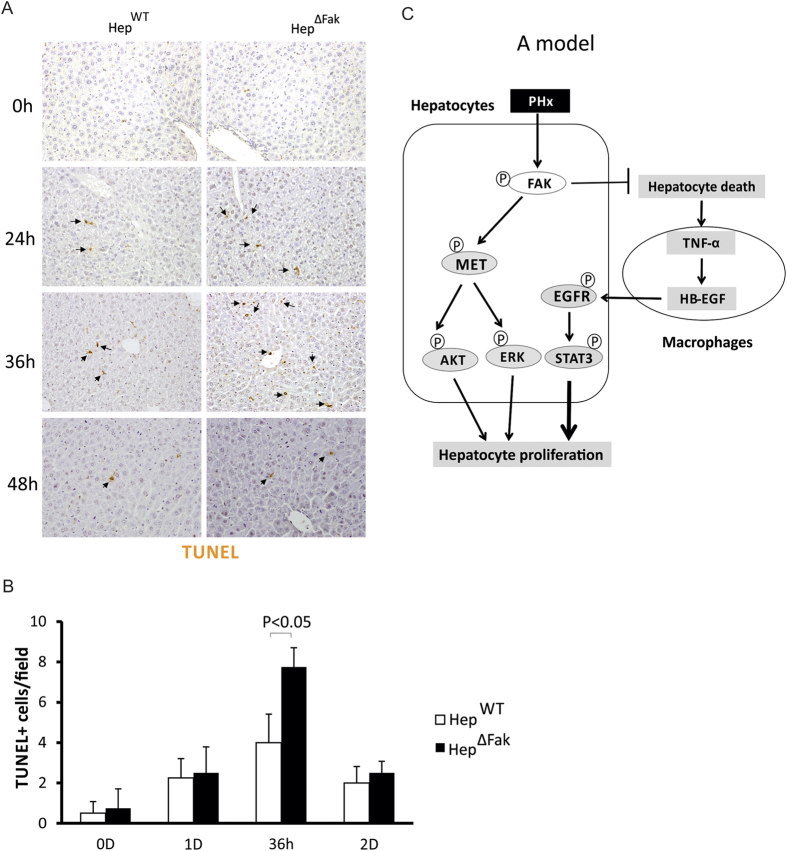
*Fak* deficiency increases hepatocyte death after PHx. **(A)** Representative pictures of TUNEL staining of livers of Hep^WT^ and Hep^∆Fak^ mice 0, 1, 1.5, and 2 days after PHx. **(B)** Quantification of TUNEL staining for **(A)** (n = 5). **(C)** A schematic diagram of the proposed mechanisms.

TNFα is mainly produced by Kupffer cells in the liver. Hepatocytes undergoing cell death release interleukin**-**1 alpha (IL-1α), which can activate Kupffer cells to produce cytokines and growth factors, including TNFα^28^,^29^,^30^,^31^. FAK plays an important role in promoting cell survival^13^. Therefore, we hypothesized that *Fak* deletion might increase hepatocyte death, thereby activating Kupffer cells to produce more TNFα after PHx. Indeed we did find more apoptosis in *Fak*-deficient livers compared to *WT* livers on days 1 and 1.5 post-PHx (Fig. 6A,B). These results suggest that *Fak* deficiency enhances TNFα expression by increasing hepatocyte death after PHx.

### *Fak* deficiency in mouse liver does not result in compensatory expression of Pyk2 in mouse liver

Pyk2, the other member of the FAK family of cytoplasmic tyrosine kinases, shares significant sequence homology and a similar structural organization as FAK^32^. It has been shown that deletion of *FAK* can lead to increased expression of endogenous Pyk2, which compensates for Fak functions in embryonic fibroblasts, adult endothelial cells and mammary cancer stem cells^33^,^34^,^35^. We therefore examined whether *Fak* deletion results in compensatory expression of Pyk2 in mouse livers after 2/3 PHx. There were no significant changes in the expression of Pyk2 or p-Pyk2 in Hep^∆Fak^ mouse livers compared to those of Hep^WT^ mice prior to or after PHx (Fig. S2). These data suggest that F*ak* deficiency in hepatocytes does not lead to a compensatory expression of Pyk2 during liver regeneration after 2/3 PHx.

## Discussion

Understanding the molecular mechanism underlying liver regeneration is important for improving the survival rate of patients after surgical resection or reducing the amount of liver tissue required for liver transplantation. In this study, we found that *Fak* deletion in hepatocytes accelerates liver regeneration after PHx. These data suggest that FAK inhibits liver regeneration and inhibition of FAK may be a promising strategy to accelerate liver regeneration in the liver transplantation setting.

Activation of EGFR and HGF/c-MET signaling is critical for liver regeneration. We found that *Fak* deletion significantly increases EGFR activation during liver regeneration. We also found greater HB-EGF expression in *Fak*-deficient livers compared to WT livers after PHx, and inhibition of HB-EGF abrogates the enhanced EGFR and accelerated liver regeneration induced by *Fak* deletion. HB-EGF has been shown to play an important role in promoting liver regeneration^22^. These data suggest that *Fak* deletion increases EGFR activation by enhancing HB-EGF expression in the liver. HG-EGF is produced by monocytes and macrophages. HB-EGF mRNA can be induced rapidly (within 1 hour) by TNFα treatment in vascular endothelial cells^25^. In this study, we found that TNFα levels were significantly higher in *Fak*-deficient livers compared to WT livers after PHx. Inhibition of TNFα by a neutralizing antibody treatment suppressed HB-EGF mRNA expression, EGFR activation and liver regeneration in *Fak*-deficient livers. These data suggest that *Fak* deletion might accelerate liver regeneration by increasing TNFα expression. The molecular mechanism by which HB-EGF is induced by TNFα remains unclear. However, the HB-EGF promoter contains multiple putative binding sites for NF-κB and c-Jun/AP1 (Fig. S3), and these can be activated by TNFα. Therefore, it is possible that TNFα might induce HB-EGF by activating of NF-κB and c-Jun/AP1. We intend to study this hypothesis in the near future.

How TNFα production is enhanced by *Fak* deletion during liver regeneration remains unclear. However, our data indicate that there is more hepatocyte death in Fak-KO mice compared to WT mice after PHx. Because dying hepatocytes release IL-1α, which was indeed higher in Fak-KO livers compared to WT livers (data not shown), and IL-1α activates Kupffer cells to produce TNFα^28^,^29^,^30^,^31^, it is reasonable to assume that *Fak* deletion increases hepatocyte death after PHx and enhances TNFα production. FAK has been shown to play an important role in cell survival in anchorage-dependent cells by binding to the death domain of receptor-interacting protein (RIP)^36,37^. Although FAK deletion does not induce apoptosis under homeostatic conditions^14^, many cytokines, including TNFα, have been induced during liver regeneration after PHx, which may result in increased apoptosis in FAK-null cells. It has been shown that increased expression of FAK partially suppresses TNFα-induced apoptosis in intestinal epithelial cells^38^. Therefore, FAK may suppress TNFα-induced hepatocyte death after PHx and the deletion of FAK would enhance TNFα-induced hepatocyte death, resulting in increased TNFα production and activation of EGFR via HB-EGF. We also found TNFα expression was decreased in *Fak*-deficient livers 2 days after PHx, suggesting that the regulation of TNFα by deletion of *Fak* is transient. Similar patterns of hepatocyte death in WT and *Fak*-deficient livers 2 days after PHx further suggests that deletion of *Fak* enhances TNFα by increasing hepatocyte death.

The increased hepatocyte proliferation and accelerated liver regeneration are suppressed in *Fak*-deficient livers by 3 days following PHx. We found that c-MET activation during liver regeneration was significantly suppressed in *Fak*-deficient livers and the HGF/c-MET pathway plays a critical role in promoting liver regeneration. Therefore, we suggest that increased EGFR activation by *Fak* deletion is sufficient to overcome the decrease in c-MET activation, thereby accelerating liver regeneration at the early time points. However, inhibition of c-MET activation by *Fak* deletion might inhibit liver regeneration when EGFR activation is diminished 3 days after PHx. Both the positive and negative effects of *Fak* deletion in liver regeneration reach a balance and liver mass is maintained in *Fak*-deficient mouse livers after PHx (Fig. 6C). How c-MET activation is inhibited by *Fak* deletion remains unclear. c-MET directly interacts with the FERM domain of FAK and phosphorylates FAK in MEFs and HEK293 cells^39^. We previously discovered that c-MET also phosphorylates FAK in mouse liver and HCC cells^14^. We intend to undertake future studies to determine whether there is a feedback loop by which FAK also regulates the activation of c-MET in hepatocytes.

In conclusion, our study shows that *Fak* deletion accelerates liver regeneration after PHx. Inhibition of FAK may offer an effective strategy to accelerate liver regeneration. FAK inhibition also shows promise in inhibiting HCC development^14^. Therefore, inhibition of FAK might kill the proverbial two birds with one single stone: suppressing tumor cell growth and accelerating normal hepatocyte regeneration. A number of FAK inhibitors have been developed and are being studied in Phase I or Phase II clinical trials for multiple solid tumors^40,41^. These inhibitors might be useful to accelerate liver regeneration, especially in patients following liver transplantation.

## Methods

### Mice and treatments

All animals received humane care according to the “Guide for the Care and Use of Laboratory Animals” (http://oacu.od.nih.gov/ac_cbt/guide3.htm). The procedures for all animal experiments were approved by the Institutional Animal Care and Use Committee of Loyola University Chicago. The generation and breeding of the Alb-Cre and Alb-Cre; Fak^flox/flox^ mice was described previously^14^. Both Alb-Cre and Alb-Cre; Fak^flox/flox^ mice were in C57BL/6 background.

For EGFR inhibitor treatments, erlotinib hydrochloride salt (LC Laboratories, Cat# E-4007) was diluted in 6% captisol (CyDex, Inc., Lenexa, KS). Two days before 2/3 partial hepatectomy, six 8–12 week-old Alb-Cre (3 males and 3 females) and twelve 8–12 week-old Alb-Cre; Fak^flox/flox^ mice (6 males and 6 females) were administered vehicle solution (6% captisol) or 50 mg/kg erlotinib by oral gavage every day until livers were collected.

For HB-EGF inhibitor treatments, CRM197 (Fisher Scientific, Cat# 5019813) was diluted in PBS. Two days before 2/3 partial hepatectomy, six 8–12 week-old Alb-Cre (3 males and 3 females) and twelve 8–12 week-old Alb-Cre; Fak^flox/flox^ mice (6 males and 6 females) were administered a vehicle solution (PBS) or 1 mg/kg CRM197 by intraperitoneal (i.p.) injection every day until collection of the livers.

For TNFα antibody treatment, anti-mouse TNFα antibody (BioXCell, Cat# BE0058) was diluted in PBS. Two days before 2/3 partial hepatectomy, six 8–12 week-old Alb-Cre (3 males and 3 females) and twelve 8–12 week-old Alb-Cre; Fak^flox/flox^ mice (6 males and 6 females) were administered a vehicle solution (PBS) or 10 mg/kg TNFα antibody by i.p. injection every day until collection of the livers.

### Partial hepatectomy

2/3 partial hepatectomy was carried out on gender-matched 8–12 week-old Alb-Cre and Alb-Cre; Fak^flox/flox^ mice following published protocols^42,43^. Two hours before sacrificing the mice, they were injected with 100 mg/kg bromodeoxyuridine (BrdU). Livers were collected on day 0, 1, 1.5, 2, 3, 5, 7 or 14 following surgery.

### Western blotting

Western blotting was performed as previously described^14^^,44^. Primary antibodies, including those for FAK, p-FAK (Y397), EGFR (Tyr 1068), EGFR, p-c-MET (Tyr 1234/1235), p-Pyk2 and p-Pyk2 were purchased from Cell Signaling (Danvers, MA). β-Actin antibody was purchased from Sigma-Aldrich. c-MET antibody was purchased from R&D systems. EGFR antibody was purchased from Santa Cruz.

### TUNEL staining

TUNEL staining was performed as previously described^14^,^44^,^45^,^46^. The apoptotic index was scored in at least 5 fields at 400× magnification/mouse and reported as mean ± SD. Five mice were used in each group.

### Immunohistochemical (IHC) staining

IHC staining was performed as previously described^14^. Cells with positive staining were scored in at least 5 fields at 400× or 200× magnification and reported as mean ± SD. Five mice were used in each group.

### Statistical analysis

Statistical analysis was carried out using GraphPad Prism V software. Data are presented as means ± standard deviatiosn (SD). Statistical significance was calculated using Student’s t test. P < 0.05 was considered to be significant. Means ± SDs are shown in the Figures where applicable.

## Additional Information

**How to cite this article**: Shang, N. *et al*. FAK deletion accelerates liver regeneration after two-thirds partial hepatectomy. *Sci. Rep.* **6**, 34316; doi: 10.1038/srep34316 (2016).

## Supplementary Material

Supplementary Information
