# Supplementary material for: FAK deletion accelerates liver regeneration after two-thirds partial hepatectomy
Source: Sci Rep. 2016 Sep 28;6:34316. doi: 10.1038/srep34316 (PMC5039626; doi:10.1038/srep34316)
Supplement: Supplementary Information [file srep34316-s1.pdf]

FAK deletion accelerates liver regeneration after two-thirds partial hepatectomy

Na Shang [1], Maribel Arteaga [1], Lennox Chitsike[1], Fang Wang[1], Navin Viswakarma [1], Peter Breslin [2,3] and Wei Qiu [1*]

Departments of [1] Surgery and Oncology Institute and [2] Molecular/Cellular Physiology, Stritch School of Medicine, 2160 South 1st Avenue, Maywood, IL 60153, USA. [3]Department of Biology, Loyola University Chicago, 1032 W. Sheridan Rd., Chicago, IL 60660, USA

Emails of authors:

NS: nshang@luc.edu; MA: marteaga@luc.edu; LC: lchitsike@luc.edu; FW: fwang6@luc.edu; NV: navinv@uic.edu; PB: pbresli@luc.edu; WQ: wqiu@luc.edu.

Key words: EGFR; c-MET; HB-EGF; TNFα; apoptosis

Disclosures: The authors have no conflicts to disclose. All authors agreed on the submission.

Author contributions:

NS performed experiments, analyzed data and wrote the paper.

MA bred mice and performed experiments.

LC, FW and NV performed experiments.

PB wrote the paper.

WQ designed experiments, analyzed data and wrote the paper.

[*]**Correspondence:**

Wei Qiu, Ph.D., 2160 South 1st Avenue., Bldg. 112 Rm. 338, Maywood, IL 60153

Email: wqiu@luc.edu; Phone: 708-327-8191; Fax: 708-327-3342

Fig. S1

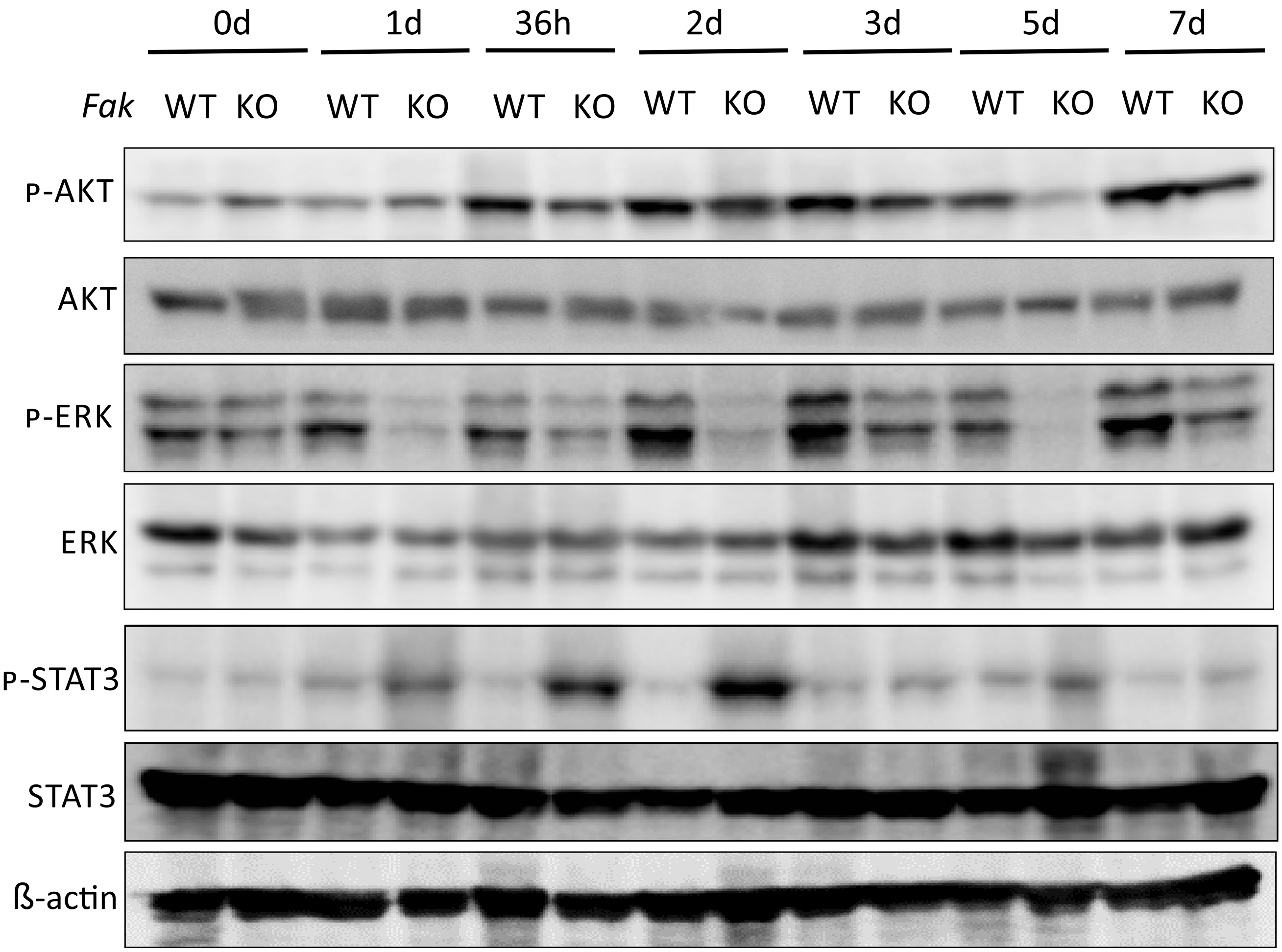
0d
1d
36h
2d
3d
5d
7d
Fak WT KO WT KO WT KO WT KO WT KO WT KO WT KO
P-AKT
AKT
P-ERK
ERK
P-STAT3
STAT3
ß-actin

Fig. S2

A

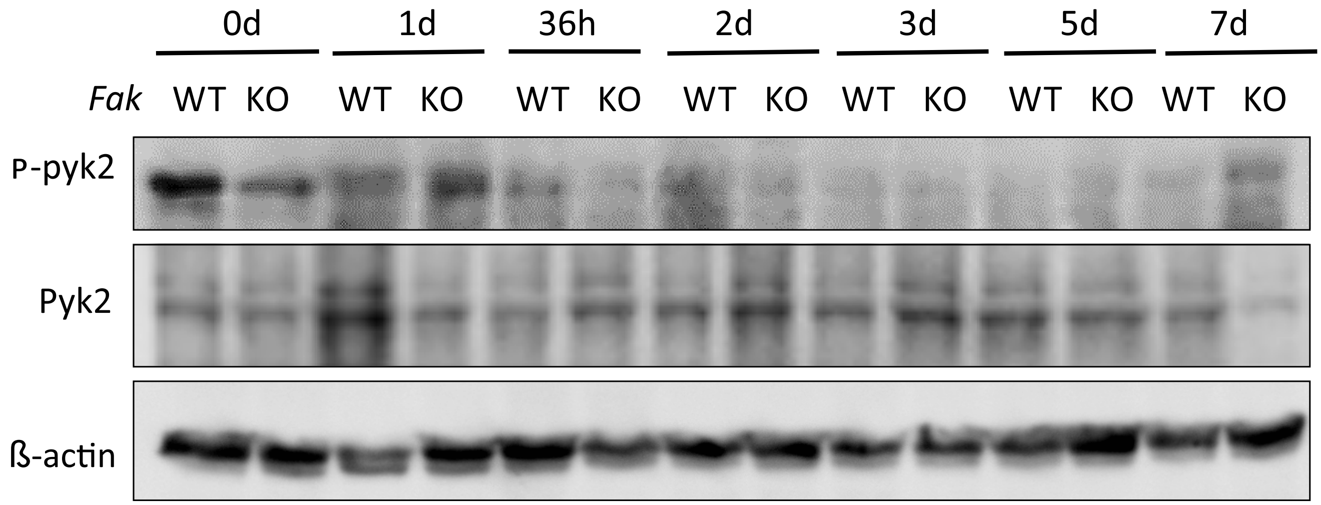
0d
1d
36h
2d
3d
5d
7d
Fak WT KO WT KO WT KO WT KO WT KO WT KO WT KO
P-pyk2
Pyk2
ß-actin

Fig. S3

Putative transcription factor binding sites in the promoter of mouse HB-EGF

The Champion ChiP Transcription Factor Search Portal

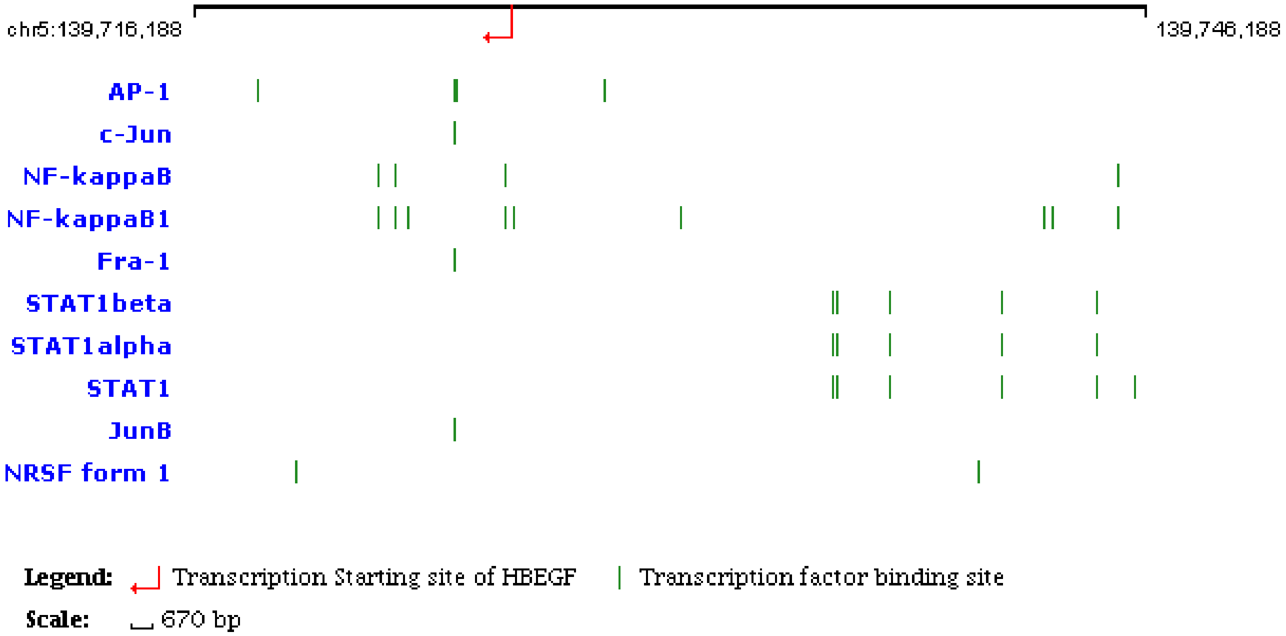

Supplemental figure legends

Figure S1. ***Fak*** **deficiency decreases phosphorylation of AKT and ERK but increases phosphorylation of STAT3 after PHx.**

Expression of p-AKT, AKT, p-ERK, ERK, p-STAT3, STAT3 and β-actin proteins in whole livers (pooled samples from 3 mice) of Hep^{WT} and $Hep^{\Delta Fak}$ mice 0, 1, 1.5, 2, 3, 5 and 7 days after PHx.

Figure S2. ***Fak*** **deficiency does not alter Pyk2 expression or activation after PHx.**

(A) Expression of p-Pyk2, Pyk2 and β-actin proteins in whole livers of Hep^{WT} and $Hep^{\Delta Fak}$ mice 0, 1, 1.5, 2, 3, 5 and 7 days after PHx.

Figure S3. Putative transcription factor binding sites in the promoter of mouse HB-EGF.

Putative transcription factor binding sites in the promoter of mouse HB-EGF was analyzed by the Champion Chip Transcription Factor Search Portal from QIAGEN.
